# Supplementary figures and images for: Cardiometabolic diseases and associated risk factors in transitional rural communities in tropical coastal Ecuador
Source: PLoS One. 2024 Jul 18;19(7):e0307403. doi: 10.1371/journal.pone.0307403 (PMC11257341; doi:10.1371/journal.pone.0307403)

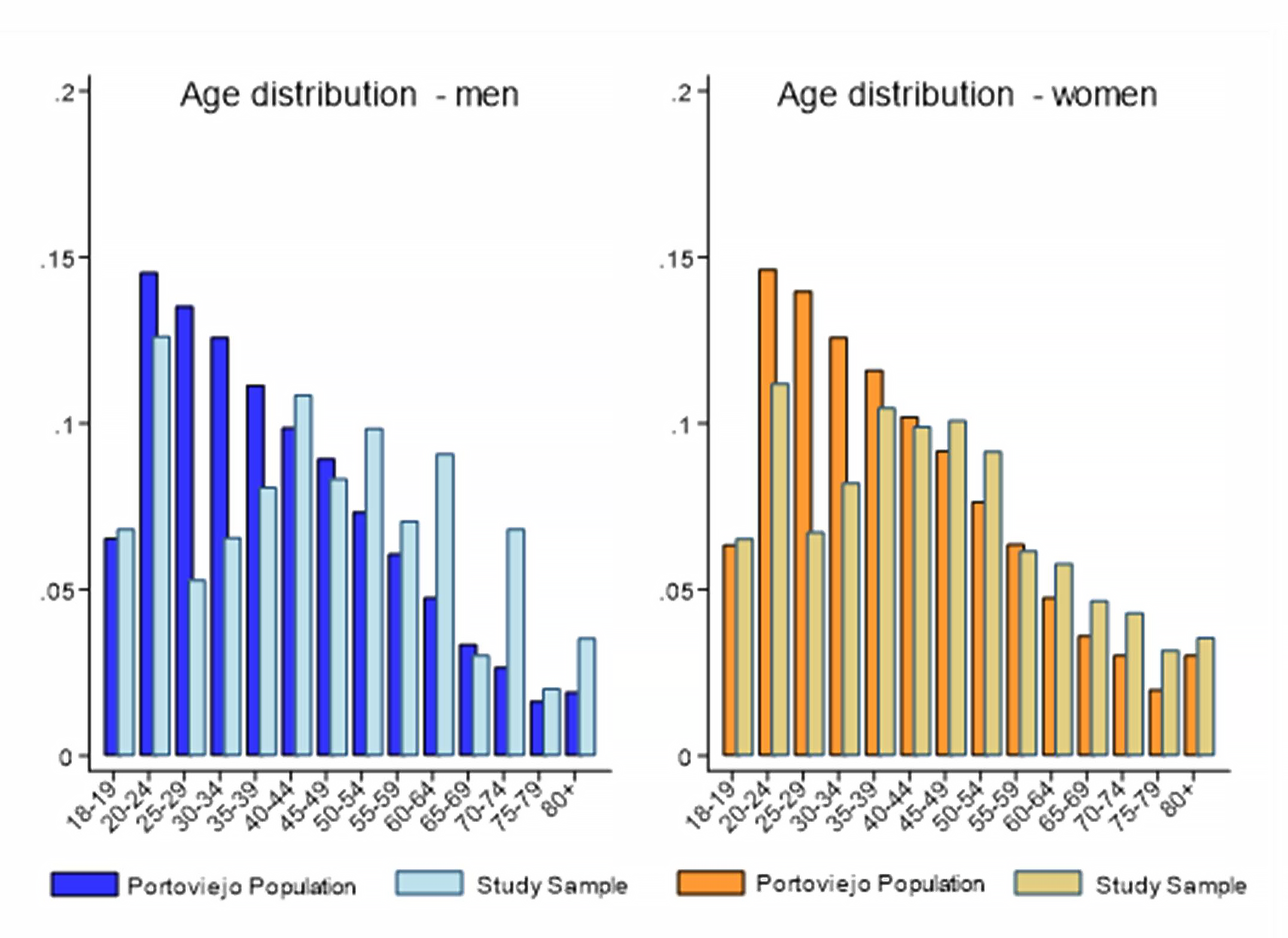

Supplement: S1 Fig — Data were obtained from the 2010 census [9]. (TIF) [file pone.0307403.s001.tif]
