# Supplementary material for: Cardiometabolic diseases and associated risk factors in transitional rural communities in tropical coastal Ecuador
Source: PLoS One. 2024 Jul 18;19(7):e0307403. doi: 10.1371/journal.pone.0307403 (PMC11257341; doi:10.1371/journal.pone.0307403)
Supplement: S1 Table — (DOCX) [file pone.0307403.s002.docx]

**S1 Table. Clinical history of diabetes and presence of hyperglycemia (Hb1Ac>=6.5%) at time of survey**

| Glycaemia | Clinical history of diabetes | | | | | |
| --- | --- | --- | --- | --- | --- | --- |
|  | All (N=927) | | Women (N=534) | | Men (N=393) | |
|  | No  (n=795) | Yes (n=132) | No  (n=440) | Yes  (n=94) | No  (n=355) | Yes  (n=38) |
| Euglycaemic | 684 (86.0%) | 34 (25.8%) | 377 (85.7%) | 24 (25.5%) | 307 (86.5%) | 10 (26.3%) |
| Hyperglycaemic | 111  (14.0%) | 98 (74.2%) | 63  (14.3%) | 70 (74.5%) | 48  (13.5%) | 28 (73.7%) |
