# Supplementary material for: Cardiometabolic diseases and associated risk factors in transitional rural communities in tropical coastal Ecuador
Source: PLoS One. 2024 Jul 18;19(7):e0307403. doi: 10.1371/journal.pone.0307403 (PMC11257341; doi:10.1371/journal.pone.0307403)
Supplement: S2 Table — (DOCX) [file pone.0307403.s003.docx]

**S2 Table. Clinical history of hypertension and presence of elevated blood pressure (>140/90 mm Hg) at time of survey**

| Blood pressure | Clinical history of hypertension | | | | | |
| --- | --- | --- | --- | --- | --- | --- |
|  | All (N=927) | | Women (N=534) | | Men (N=393) | |
|  | No  (n=623) | Yes (n=304) | No  (n=335) | Yes  (n=199) | No  (n=288) | Yes  (n=105) |
| Normal | 513 (82.3) | 106 (34.9) | 288 (86.0) | 74 (37.2) | 225 (78.1) | 32 (30.5) |
| Elevated | 110 (17.7) | 198 (65.1) | 47 (14.0) | 125 (62.8) | 63 (21.9) | 73 (69.5) |
