# Supplementary material for: Cardiometabolic diseases and associated risk factors in transitional rural communities in tropical coastal Ecuador
Source: PLoS One. 2024 Jul 18;19(7):e0307403. doi: 10.1371/journal.pone.0307403 (PMC11257341; doi:10.1371/journal.pone.0307403)
Supplement: S3 Table — Estimates and 95% confidence intervals (CI) were derived from linear regression analyses and accounted for household and community clustering and were weighted for population structure. Associations, i.e. the adjusted mean differences between groups defined by indicators of adiposity were adjusted for the sociodemographic variables shown in the Table (including age and age2 [all outcomes], age3 [HbA1c and fasting glucose], sex [all outcomes], and the interaction between age and sex [all outcomes]). Abbreviations: BP–blood pressure; WHtR–waist-to-height ratio; VAI–Visceral adiposity index. Definitions for indicators of adiposity: Overweight (BMI≥25); abdominal obesity (waist circumference of ≥94 cm in men and ≥88 cm in women); increased body fat (men (≥25%, women (≥30%); WHtR (≥0.5); elevated VAI (age <30 –VAI >2.52; age ≥30 & <42 –VAI > 2.23; age ≥42 & <52 –VAI >1.92; age ≥52 & <66 –VAI > 1.93; age ≥66 –VAI > 2). Missing data: increased fat (205). (DOCX) [file pone.0307403.s004.docx]

**S3 Table. Age-and sex adjusted associations between indicators of adiposity and systolic and diastolic blood pressure (mm Hg), glycosylated haemoglobin (HbA1c) (%), and fasting glucose (mg/dL) in 930 adults.**

| **Variable** | **Systolic BP** | | **Diastolic BP** | | **HbA1c** | | **Fasting glucose** | |
| --- | --- | --- | --- | --- | --- | --- | --- | --- |
|  | **Estimate (95% CI)** | **P value** | **Estimate (95% CI)** | **P value** | **Estimate (95% CI)** | **P value** | **Estimate (95% CI)** | **P value** |
| **Socio-demographic** | | | | | | | | |
| Age | **0.911 (0.819, 1.002)** | **<0.001** | **0.34 (0.288, 0.392)** | **<0.001** | **0.035 (0.026, 0.044)** | **<0.001** | **1.328 (0.874, 1.328)** | **<0.001** |
| Age^2^ | **-0.004 (-0.007, -0.001)** | **0.028** | **-0.009 (-0.011, -0.006)** | **<0.001** | -0.00008 (-0.0004, 0.0002) | 0.670 | -0.005 (-0.015, 0.004) | 0.258 |
| Age^3^ | ---------------------- | **--------** | ---------------------- | **--------** | -**0.00002 (-.00004, -8.95e-06)** | **0.001** | **-0.001 (-0.001, -0.00001)** | **0.004** |
| Sex (Male vs. Female) | **2.237 (-0.199, 4.672)** | **0.072** | 0.043 (-1.459, 1.545) | 0.955 | **-0.408 (-0.619, -0.197)** | **<0.001** | **-9.53 (-15.39, -3.671)** | **0.001** |
| Interaction: Age × Sex | **-0.451 (-0.589, -0.313)** | **<0.001** | -**0.116 (-0.197, -0.035)** | **0.005** | **-0.018 (-0.029, -0.007)** | **0.001** | **-0.523 (-0.825, -0.221)** | **0.001** |
| **Indicators of adiposity** | | | | | | | | |
| Overweight (Yes vs. No) | **9.848 (7.391, 12.305)** | **<0.001** | **6.392 (4.874, 7.911)** | **<0.001** | **0.354 (0.179, 0.530)** | **<0.001** | **8.831 (4.409, 13.253)** | **<0.001** |
| WHtR elevated (Yes vs. No) | **7.118 (3.764, 10.472)** | **<0.001** | **5.257 (3.127, 7.387)** | **<0.001** | **0.337 (0.207, 0.467)** | **<0.001** | **8.471 (5.342, 11.601)** | **<0.001** |
| Abdominal obesity (Yes vs. No) | **9.44 (7.114, 11.766)** | **<0.001** | **6.551 (4.934, 8.169)** | **<0.001** | **0.441 (0.259, 0.623)** | **<0.001** | **10.399 (6.03, 14.767)** | **<0.001** |
| Increased body fat (Yes vs. No) | **7.884 (0.732, 15.036)** | **0.031** | **5.372 (1.662, 9.081)** | **0.005** | **0.369 (0.121, 0.618)** | **0.004** | **11.903 (5.991, 17.815)** | **<0.001** |
| High VAI (Yes vs. No) | **4.047 (1.333, 6.762)** | **0.004** | 1.613 (-0.113, 3.338) | 0.067 | **0.411 (0.228, 0.593)** | **<0.001** | **11.365 (6.651, 16.079)** | **<0.001** |

Estimates and 95% confidence intervals (CI) were derived from linear regression analyses and accounting for household and community clustering and weighted for population structure. Associations, i.e. the adjusted mean differences between groups defined by indicators of adiposity were adjusted for the sociodemographic variables shown in the Table (including age and age^2^ [all outcomes], age^3^ [HbA1c and fasting glucose], sex [all outcomes], and the interaction between age and sex [all outcomes]). Abbreviations: BP – blood pressure; WHtR – waist-to-height ratio; VAI – Visceral adiposity index.

Definitions for indicators of adiposity: Overweight (BMI≥25); abdominal obesity (waist circumference of ≥94 cm in men and ≥88 cm in women); increased body fat (men (≥25%, women (≥30%); WHtR (≥0.5); elevated VAI (age <30 – VAI >2.52; age ≥30 & <42 – VAI > 2.23; age ≥42 & <52 – VAI >1.92; age ≥52 & <66 – VAI > 1.93; age ≥66 – VAI > 2). Missing data: increased fat (205).
